# Clinical presentation of late-onset psychosis (LOP) and differential diagnosis with dementia: a case report

**DOI:** 10.1192/j.eurpsy.2023.2274

**Published:** 2023-07-19

**Authors:** S. Oller, R. Esteve Vila, M. Pérez Machado

**Affiliations:** psychiatry, Hospital Del Mar, BARCELONA, Spain

## Abstract

**Introduction:**

Late-onset psychosis appears in people over the age of 40. Some preliminary studies show that LOP has fewer severe positive symptoms, more systematic persecutory delusions, more bizarre-type delusions, less affective flattening, and more social withdrawal than early onset psychosis.

There are some studies that consider late-onset and very late-onset psychosis as prodromes of neurodegenerative disease. There are some differences in neuropsychological profiles and specific cognitive function alterations discovered. More evidence, however, is required to make an accurate diagnosis.

**Objectives:**

The objective of this study was to reflect the difficulties in differentiating between late-onset psychosis and dementia by reporting the case of a 77-year-old woman who presented with mystical-religious delusions and hallucinations during her hospitalization.

**Methods:**

We present the case of a 77-year-old woman who was hospitalized because of a stroke. During her stay, she began receiving follow-up from the mental health team because she verbalized some mystical-religious delusional ideas. During the psychiatric interview, the patient verbalized mystical-religious ideas and oscillated between coherent, organized, and disaggregated speech. No problems were detected with orientation, or florid affective symptoms that could point to a delirium or affective disorder. The premorbid personality was extravagant, with interpersonal difficulties and magical thinking. Nonetheless, she had no prior contact with the mental health system or hospitalization. We could approximate the beginning of the symptomatology at around 60 years old, thanks to her relatives. Prior to this age, she maintained good function by working as a chef on a regular basis. She gradually isolated herself due to her lack of mobility. Similarly, she decreases her self-care activities, begins hoarding items around the house, and gradually develops more psychotic symptoms.A brain scan was performed, and no acute pathology was found. A neuropsychological test was not executed due to a lack of collaboration from the patient.

**Results:**

-

**Conclusions:**

This case reflects the complexity of differentiating between dementia and late-onset psychosis. Supplementary testing and follow-up are essential for establishing a diagnosis. Related to that, more research is needed to identify the differential characteristics between the two disorders and the temporal correlation between them.

**Disclosure of Interest:**

None Declared

